# Fracture toughness of zirconia with a nanometer size notch fabricated using focused ion beam milling

**DOI:** 10.1002/jbm.b.34668

**Published:** 2020-06-20

**Authors:** Yifeng Liao, Max Gruber, Henry Lukic, Si Chen, Spiro Megremis

**Affiliations:** ^1^ American Dental Association Science & Research Institute, LLC Chicago Illinois USA; ^2^ Advanced Photon Source Argonne National Laboratory Lemont Illinois USA

**Keywords:** focused ion beam, fracture toughness, grain size, notch, phase transformation, SEVNB, zirconia

## Abstract

**Objectives:**

Zirconia with 3 mol% yttria (3Y‐TZP) has been used for dental crowns and bridges due to its excellent mechanical behavior. Performing fracture toughness testing on this nanograin material, however, can be a challenge. For reliable results, fracture toughness testing requires an extremely sharp notch in the test specimen that closely approximates a very sharp crack. This study was to investigate an alternative method to produce nanometer‐sized notches, which are less than the average grain size of 3Y‐TZP, during the preparation of single‐edge V‐notched beam specimens and report the resulting fracture toughness value.

**Methods:**

We present a method using focused ion beam (FIB) milling to fabricate nanometer‐sized notches in 3Y‐TZP. The notch tip is <100 nm wide, which is smaller than the grain size, and is consistent throughout the thickness of the specimen.

**Results:**

The FIB‐notched specimens show a much reduced average fracture toughness of 5.64 ± 1.14 MPa√m compared to 8.90 ± 0.23 MPa√m for the specimens without FIB‐notches. The FIB‐milling did not appear to create any monoclinic phase prior to fracture toughness testing. Fractures originated at the FIB‐notches, and the notch size can be readily identified post‐mortem using a microscope. A considerable amount of tetragonal‐to‐monoclinic phase transformation was observed throughout the fracture surfaces.

**Significance:**

FIB milling provides an alternative method to fabricate nanometer‐sized notches that are smaller than the grain size of tetragonal zirconia polycrystal. The fracture toughness determined using FIB‐notches was ~5.64 MPa√m, smaller than the specimens with V‐notches fabricated using saw blades.

## 1 INTRODUCTION

Dental crowns and bridges made of zirconia have gained remarkable popularity due, in part, to their resistance to catastrophic failure (Bindl & Mörmann, 2002; Conrad, Seong, & Pesun, 2007; Denry & Kelly, 2008; Esquivel‐Upshaw, Anusavice, Young, Jones, & Gibbs, 2004; Fradeani, Aquilano, & Corrado, 2002; Fradeani, D'Amelio, Redemagni, & Corrado, 2005; McLaren & White, 2000; Miyazaki, Nakamura, Matsumura, Ban, & Kobayashi, 2013; Raigrodski et al., 2006; Sorensen, Choi, Fanuscu, & Mito, 1998; von Steyern, Carlson, & Nilner, 2005; Wolfart, Bohlsen, Wegner, & Kern, 2005). Tetragonal zirconia polycrystal stabilized with 3 mol% of yttria (3Y‐TZP) preserves the metastable tetragonal phase at room temperature (Zhao & Vanderbilt, 2002), and provides superior strength and fracture toughness compared to other biomedical ceramic materials (Piconi & Maccauro, 1999). Fracture toughness value essentially represents a material's intrinsic resistance to crack extension in mode I fracture. Unlike flexural strength, which can vary with specimen size and large defects, fracture toughness is a material property less influenced by post‐processing and handling of the material.

Extensive efforts have been documented to improve fracture toughness measurement methods for dental ceramics (Kelly & Denry, 2008; Piconi & Maccauro, 1999); however, fracture toughness measurements for zirconia, and brittle materials in general, are nontrivial. A general difficulty lies in the fact that a sharp precrack is required to achieve reliable results. Fenghui (2000) showed that fracture toughness reaches a constant value when the notch‐tip radius is reduced to the grain diameter for notched alumina materials. Therefore, the width of the notch tip should be sharpened to a submicron level in order to conduct a convincing fracture toughness test for submicron‐grained zirconia.

Three methods for creating sharp cracks in beam specimens for determination of fracture toughness of advanced ceramics are introduced in ASTM C 1421 (ASTM, 2018), including the single‐edge pre‐cracked beam (SEPB) and the surface crack in flexure (SCF) methods. Using a bridge‐loading technique, the SEPB method extends precracks from either a shallow sawed notch or indentations (Jodha, Marocho, & Griggs, 2018; Quinn, Kübler, & Gettings, 1994). The geometry and residual deformation at the tip of the precrack influence the measurement, which should be treated with caution when calculating the fracture toughness value (Suresh, Ewart, Maden, Slaughter, & Nguyen, 1987). When there is a plastic zone under the precrack, as is created in the SCF method (which exploits a Knoop indenter to induce a primary median crack), the plastic zone needs to be removed by polishing (ASTM, 2018). ISO 23146 (2008) specifies the single‐edge V‐notched beam (SEVNB) method for the fracture toughness of advanced ceramics, where the starter notch is sharpened by using a razor blade. However, this standard is not recommended for tetragonal 3Y‐TZP due to its fine grain size of a few hundred nanometers (ISO 23146, 2008). The standard includes analysis of an interlaboratory evaluation of the SEVNB fracture toughness test procedures (Kübler,1999 2000), which found a notch‐root radius dependence for the fracture toughness of 3Y‐TZP and suggested that the participants of the study were not able to achieve a sufficiently sharp notch‐root radius. For the SEVNB method to be comparable to other standard methods, such as the SEPB and SCF methods, it is critical to create a notch‐tip that closely approximates a sharp crack (ISO 23146, 2008). For instance, for the fracture toughness value of 3Y‐TZP obtained from the SEVNB method to compare to the value from the SCF method, it has been suggested that the notch width must be less than about twice the size of a major microstructural feature (Kübler, 1999, 2000). Testing on partially stabilized zirconia showed that fracture toughness values from the SEVNB method agree with SEPB data as long as the notch root radii for the SEVNB specimens are <5–7 μm (Gogotsi, 1998). ISO 6872 (2015), which is specific to dental ceramic materials, also notes that because the pre‐notch made by the SEVNB method is larger than about 1 μm, alternative fracture toughness methods are recommended for use with 3Y‐TZP.

Considering the documented difficulties of machining a notch‐tip radius of sufficient sharpness in 3Y‐TZP for the SEVNB method, we report an examination of the use of focused ion beam (FIB) milling to create nanometer sized notches in standard SEVNB specimens of the material. FIB focuses an energized ion source down to nanometers, and is capable of removing small volumes of materials at specified locations. Fett, Creek, Wagner, Rizzi, & Volkert, (2008) originally reported fracture toughness test results from FIB‐notched Ce‐doped zirconia specimens with a grain size of ~1.7 μm. In their pioneering FIB study, two trapezoidal shaped bend bars were prepared with FIB‐notches of ~40 μm in depth introduced into the tensile side of each bar, which had a thickness of about 0.5 mm. With the rapid progress of instrumentation, FIB systems have become more and more accessible in both academia and industry for precision, nanometer scale materials processing. Therefore, this study was to consider the use of FIB milling, during the preparation of standard single‐edge V‐notched beam specimens in 3Y‐TZP material, to produce nanometer‐sized notches that are less than the average grain size of the material and report the resulting fracture toughness value.

## 2 MATERIALS AND METHODS

Eleven test specimens were milled from BruxZir Solid zirconia milling blanks (Lot# BZ0014191). A V‐shaped notch with a depth of ~2.25 mm was fabricated at the center of each specimen using a modified saw blade technique (Megremis et al., 2010). Briefly, this technique uses a tungsten‐molybdenum high‐speed steel, slotting saw (Thurston Manufacturing Co., Part No. J‐35) that is sharpened with an 80 grit surface grinding wheel to produce a cutting tip with a 10° included angle. The modified slotting saw was then used to machine a V‐notch in the green state specimen. Note that Krell (1994) prepared notches prior to sintering, in green state 3Y‐TZP, and showed that this method eliminated residual stresses and led to a reliable measurement.

After milling and notching, the specimens were sintered at 1,580°C for 2.5 h using a Shenpaz SintraPlus furnace following the milling blank manufacturer's instructions. During the sintering process, the specimens were placed on a layer of zirconia beads to minimize warping and contamination from the alumina‐containing crucible. Note that each milling block was labeled with an enlargement factor; therefore, the test specimens were milled oversized according to this enlargement factor. After sintering, the final size of the specimens was 3 mm × 4 mm × 45 mm with V‐shaped notches of ~1.8 mm.

Six of the 11 specimens had an additional notch milled into the center of the V‐notch using FIB‐milling. First, the specimens were sputter coated with an ~12 nm layer of gold to ensure good conductivity. Next, fine notches were milled at the center of the tip of the V‐notch for each of the specimens using a Zeiss NVision FIB system equipped with both ion beam microscopy and scanning electron microscopy (SEM). The FIB‐notch was milled using a gallium ion beam at a rate of ~25 μm per min at 30 kV with a milling current of 13 nA. The average time for milling one specimen was ~2 h. In order to examine the influence of the irradiation, a 200 μm × 200 μm square was milled in a piece of zirconia block at 30 kV and 13 nA for 2 h.

All of the test specimens were then tested using an Instron 5582 universal test system with a crosshead speed of 0.5 mm/min. Fracture toughness was determined using a four‐point bending configuration using equations described in ISO 23146 and ASTM C 1421 (ASTM, 2018), with the upper span 20 mm and the lower span 40 mm.

The fracture surfaces were examined using a Nikon stereo microscope, a Zygo NewView 8300 optical interferometer, and a JEOL JCM‐6000 benchtop scanning electron microscope. A Horiba XploRA Plus confocal Raman microscope system was used to collect site‐specific Raman spectra from fracture surfaces and FIB‐notches, as well as undeformed surfaces. A 532 nm laser source was used to activate the Raman spectra.

At the Advanced Photon Source (APS) of the Argonne National Laboratory (ANL), X‐ray fluorescence microscopy (XRF) was performed at both the 2‐ID‐D beamline and the Bionanoprobe (Chen et al., 2014) at the 9‐ID‐B beamline to obtain chemical composition. The incident X‐ray energy was tuned to 20 keV using a double‐crystal Si(111) monochrometer. A silicon drift energy dispersive detector was placed at 90° with regards to the incident beam to collect the XRF signal. For each specimen, a 250 μm × 250 μm area was surveyed with a 25 s exposure time, yielding an average chemical composition. Spectrum fitting and quantification were performed using a software program named MAPS (Vogt, 2003).

## 3 RESULTS

**FIGURE 1:**
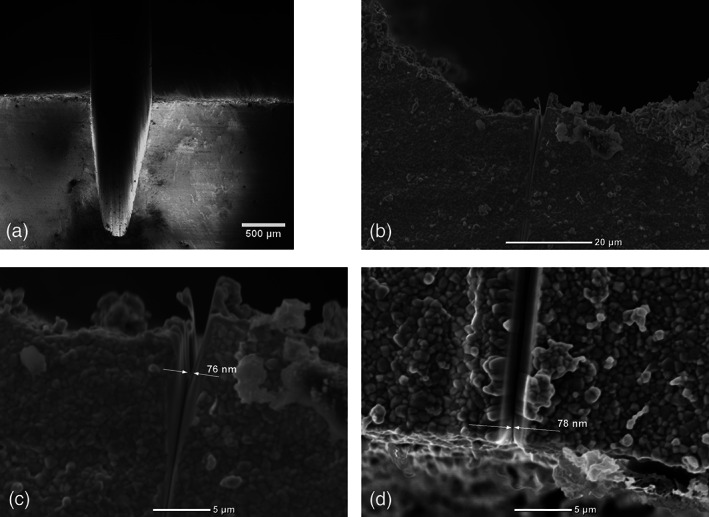
SEM images of the V‐notch with additional FIB‐notch milled into the center. (a) Overview of the V‐notch. The FIB‐notch is located at the center of the V‐notch cut by the saw blade. (b,c) Side views of the FIB‐notch at higher magnifications. The tip of the FIB‐notch is ~76 nm, much smaller than the grain size. (d) Top view of the V‐notch with FIB‐notch in the center. The tip of the FIB‐notch is ~78 nm, measured from the top‐view. The grain size is ~300 nm

The XRF analysis showed that the sintered specimens were comprised of 2.4 mol% yttria and trace amounts of calcium, iron, cobalt, and copper compounds. Due to the detection limitation, aluminum and lighter elements were not included in this XRF compositional analysis. Figure 1 shows a series of SEM images of a V‐notch with a FIB‐notch milled in the center. Figure 1b shows a high magnification image of the V‐notch machined in the specimen, and it can be seen that the notch width measures ~150 μm across its opening. Centrally located at the base of the saw blade‐notch is the milled FIB‐notch, which tapers down to an ~76 nm diameter tip, as shown in Figure 1c,d (side view and top view). The grain size of the zirconia specimen is ~300 nm, as can be seen in Figure 1c,d. From the series of images in Figure 1, it is evident that the FIB‐notch tip features a much smaller size than the grains. For all specimens, no significant cracks or other damage were found at the base of the saw blade‐notch except some occasional pores remaining from the blank manufacturing process.

Table 1 lists the fracture toughness values and FIB depths for the six FIB‐milled specimens, samples #1–6. The average fracture toughness of the FIB‐notched specimens was 5.64 ± 1.14 MPa√m, which is lower than for the specimens with saw blade‐notches only (samples #7–11). These latter specimens had an average fracture toughness value of 8.90 ± 0.23 MPa√m. One of the FIB‐notched specimens (#3) appeared to be an outlier, with a higher fracture toughness value of 8.00 MPa√m, while the rest of the specimens were between 4.60 and 6.00 MPa√m. Further investigation is needed to understand the origin of the high fracture toughness for this outlying specimen.

**TABLE 1:** Fracture toughness values of 3Y‐TZP

Sample #	Fracture toughness (MPa√m)	FIB‐notch depth (μm)
1	4.60	5.91
2	5.35	5.00
3	8.00	5.23
4	4.82	5.00
5	6.00	3.86
6	5.07	4.85
7	9.02	–
8	9.29	–
9	8.43	–
10	8.98	–
11	8.78	–

**FIGURE 2:**
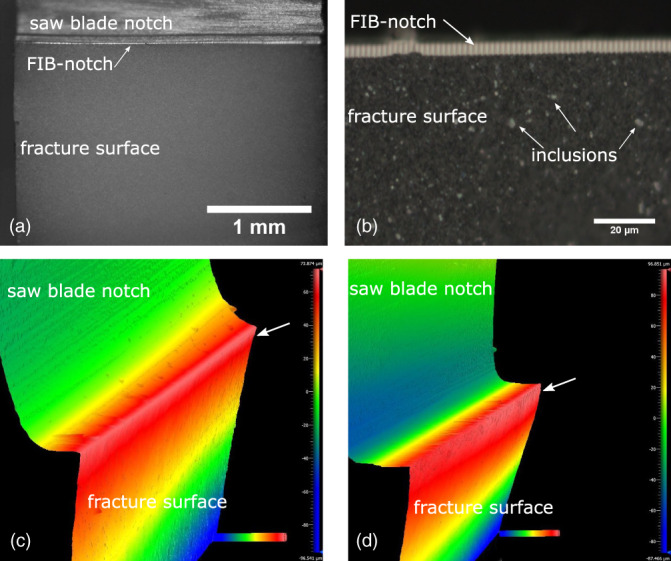
(a, b) Optical images of the fracture surface of a FIB‐notched specimen. Fracture started from the FIB‐notch, which shows bright contrast. A large amount of bright spots are present in the fracture surface. (c) Surface topography of the fracture surfaces of a FIB‐notched specimen showing smooth surface near the FIB‐notch (arrowed). (d) Surface topography of a fracture specimen with saw blade‐notch only. A strip of ridges and groves is present at the fracture surface (arrowed)

All specimens showed relatively smooth fracture surfaces. Figure 2a shows an optical image of the fracture surface from a FIB‐notched specimen. The FIB‐notched surface shows a strip of bright contrast due to its high degree of smoothness. No ridges or excessive dimples, which would indicate a brittle nature, were observed in the fracture surface. There was no discernable subcritical crack growth zone, and examination of the load/displacement curves for the specimens also did not indicate signs of subcritical crack growth. Figure 2b is a high magnification image of a region of the fracture surface shown in Figure 2a, showing greater detail of the FIB‐notch and fracture surface. The FIB‐notch depth, ranging from 3.86 to 5.91 μm (see Table 1), can be readily identified post‐mortem due to its distinct contrast. The fracture originated from the FIB‐notch, and the fracture surface features a large number of inclusions throughout, as shown in Figure 2b. These inclusions featured irregular shapes in bright contrast, with sizes ranging from 0.5 to 5 μm.

Figure 2c,d show the topography of the fracture surfaces imaged using an optical interferometer. The height of each specimen is color‐coded. In Figure 2c, the arrowed fracture surface near the FIB‐notch is relatively smooth, in contrast to the rough surface at the fracture origin for the saw blade‐notch only specimen, as arrowed in Figure 2d. For the FIB‐notched specimens, the surface roughness of the regions within 10 μm from where the cracks were initiated was measured to be 0.234 μm ± 0.069 μm. For comparison, the fracture surfaces were slightly curved for the specimens without a FIB‐notch, and the surface roughness within 10 μm from the crack initiation for the saw blade‐notched specimens was 0.404 μm ± 0.025 μm. Over 100 μm away from the crack initiation sites, the fracture surface roughness for both the FIB‐notched and saw blade‐notched specimens converged to ~0.458 μm.

**FIGURE 3:**
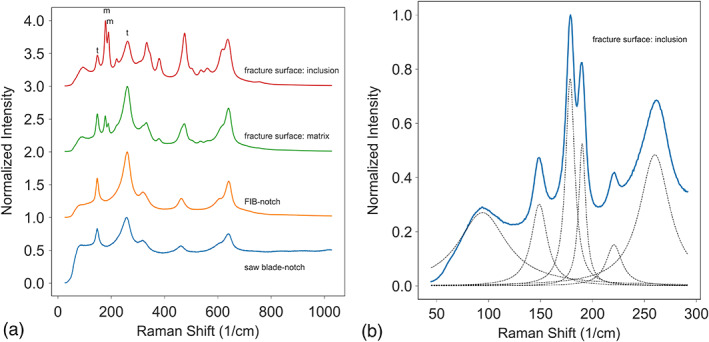
(a) Raman spectra from different locations of the fracture surfaces. The m and t refer to monoclinic and tetragonal peaks, respectively. The saw blade‐notch and FIB‐notch surfaces are tetragonal phases. The fracture surface contains 29% monoclinic phase in the matrix, and the monoclinic phase increased to 64% at the bright inclusions. The saw blade‐cut has a high background due to the gold coating. (b) The spectrum of a bright inclusion fitted with tetragonal and monoclinic peaks

The fracture surfaces of the specimens were further examined using a confocal Raman microscope in order to determine any phase changes that may have occurred as a result of the propagating cracks. At least nine spectra were collected from each specimen. Figure 3a shows the Raman spectra collected from four surface locations on a single specimen: the fracture surface; a bright inclusion in the fracture surface; the FIB‐notched surface; and the saw blade‐notched surface. The Raman spectra for the other specimens were consistent with what is shown in Figure 3a. Both the FIB‐notched and saw blade‐notched surfaces showed typical tetragonal zirconia bands (Ishigame & Sakurai, 1977; Phillippi & Mazdiyasni, 1971) at 148 and 264 cm^−1^ with no other peaks between them. In contrast, the fracture surfaces showed additional peaks at 181 and 192 cm^−1^, which are associated with zirconia's monoclinic phase. The fraction of monoclinic phase with respect to the entire zirconia content is determined by comparing the intensity of the Raman peaks according to the following equation (Chevalier, 2006; Clarke & Adar, 1982; Lughi & Sergo, 2010).

$$X_{m}=\frac{\;I_{m}^{181}+I_{m}^{192}}{I_{m}^{181}+I_{m}^{192}+0.97\;\left(I_{t}^{148}+I_{t}^{264}\right)}$$

where *I*_*m*_ and *I*_*t*_ refer to the monoclinic and tetragonal peak intensities, respectively.

Figure 3b shows the Raman spectrum of a bright inclusion in the range of 45–290 cm^−1^. The fine dashed‐line curves are the fitted constituent peaks. It is calculated that the matrix of the fracture surface is comprised of ~29% monoclinic phase; however, the region of the bright inclusion is ~64% monoclinic. No discernible monoclinic phase was detected in either of the FIB‐notched or saw blade‐notched regions.

**FIGURE 4:**
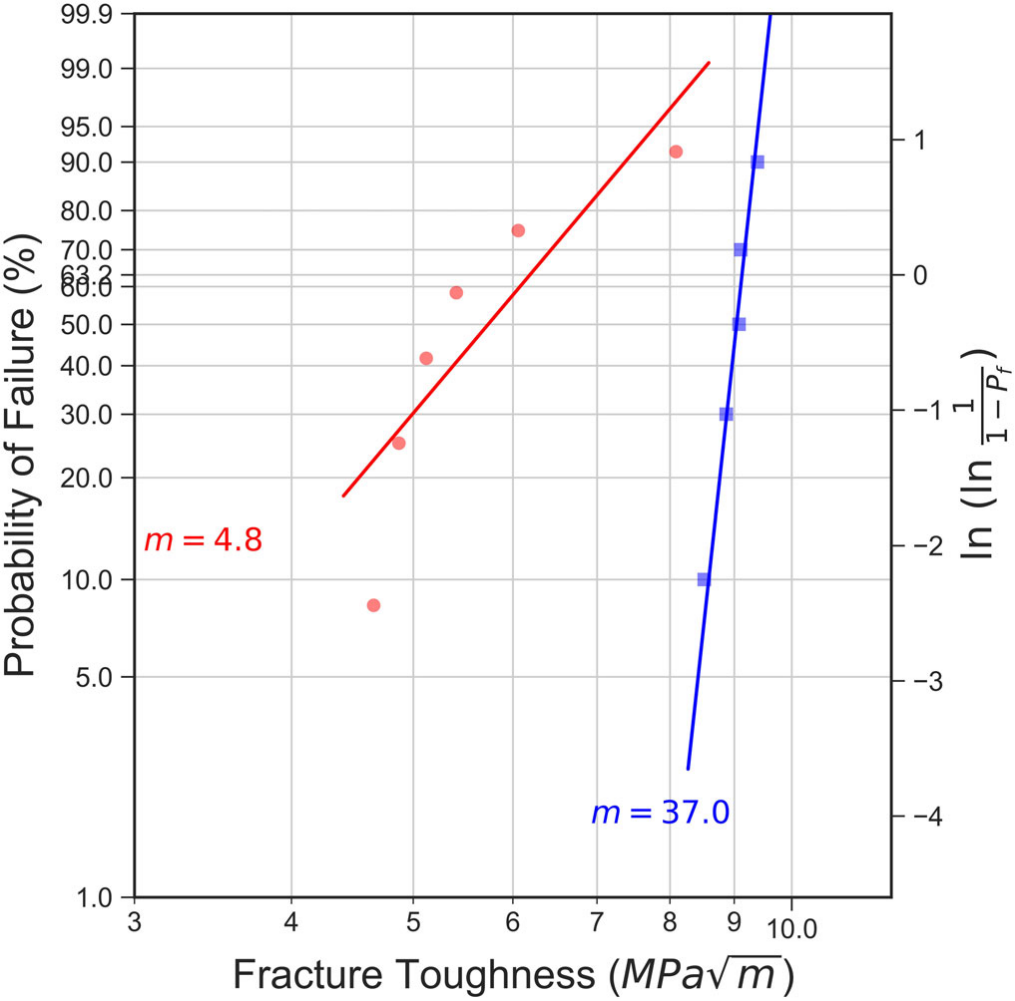
Weibull plot of fracture toughness. The Weibull moduli for the FIB‐notch (circle) and saw blade‐notch (square) are fitted to be 4.8 and 37.0, respectively

The Weibull plots of the fracture toughness values are shown in Figure 4. The fracture toughness values for the blade‐notched specimens (squares) had consistently higher values, and the Weibull modulus was calculated to be 37.0. The FIB‐notched specimens (circles) demonstrated considerably reduced fracture toughness values. Due to one outlier, the Weibull modulus for the FIB‐notched specimens was calculated to be 4.8. Further testing with more specimens is needed to provide additional insights into the variability of the mechanical behavior of zirconia.

**FIGURE 5:**
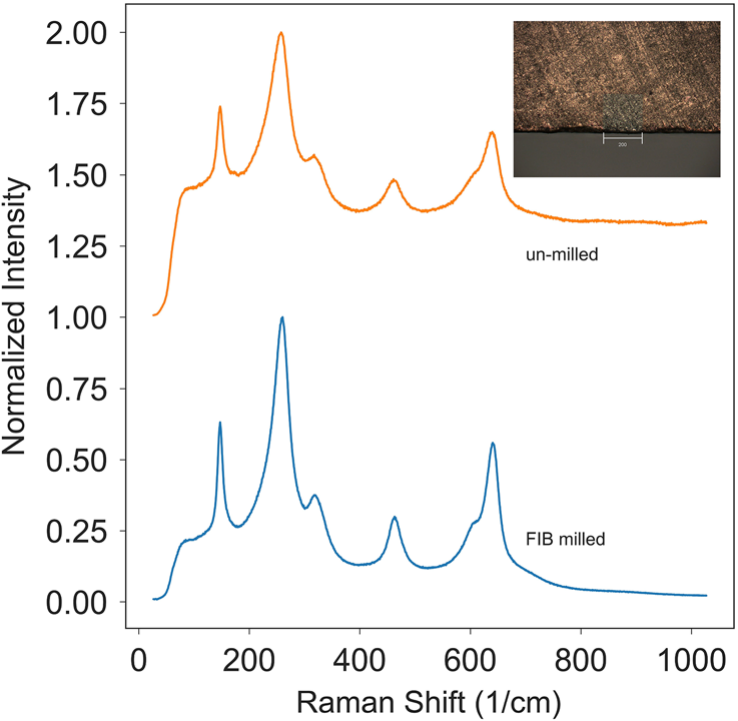
Raman spectra of FIB‐milled and un‐milled zirconia surfaces. Un‐milled surfaces were sputter coated with a thin layer of gold for conductivity. The inset shows a 200 μm × 200 μm square, FIB‐milled at 13 nA for 2 h. The FIB‐milling did not induce a discernible amount of monoclinic phase

Zirconia requires a relatively high milling current (13 nA in this study) to effectively fabricate a through‐thickness notch. For tetragonal zirconia, it is necessary to understand whether such ion beam milling alters the microstructure, particularly the phase transformation from the tetragonal to monoclinic structure. Figure 5 shows a 200 μm × 200 μm square, FIB‐milled at 13 nA for 2 h. The Raman spectra appear similar for both the FIB‐milled (blue) and un‐milled (orange) areas. No monoclinic phase was discerned, indicating that microscopically the ion beam milling had preserved the tetragonal structure.

## 4 DISCUSSION

Zirconia dental restorations are not immune to catastrophic failure. These restorations, comprised of nanometer‐sized grains, have pores, flaws, inclusions, and machining defects. Under load, the stress near pre‐existing flaws is concentrated and, thus, enables the flaws to extend. The stress configurations at these flaws are sensitive to flaw shape and size. To address this challenge, fracture toughness testing methodologies for 3Y zirconia have been of great interest to standards organizations and, likewise, the dental community. The SEVNB method is a standard method for determination of the fracture toughness of advanced ceramics, and the precracking technique in the method is considered to be relatively easy to carry out compared to other standard methods, as long as a sufficiently small notch‐root radius is created in the beam specimens (ISO 2008). The literature (Damani, Schuster, & Danzer, 1997; Gogotsi, 2003) suggests that the sharpness of a notch, rather than its shape, plays a critical role in determining the fracture toughness of a ceramic material. For example, using the SEVNB method, it was shown that the fracture toughness for partially stabilized zirconia decreases from ~9 MPa√m for an ~300 μm V‐notch root radius to ~5 MPa√m for an ~3 μm V‐notch root radius (Gogotsi, 2003). Furthermore, a study by Fischer et al. (Fischer, Waindich, & Telle, 2008) on the influence of the notch root radius on the fracture toughness values of 3Y‐TZP SEVNB specimens showed that for notch root radii between 167 and 18 μm, the fracture toughness values ranged between 13.6 and 5.9 MPa√m, respectively. In the current study, we observed similar decreases from an average of 8.90 MPa√m for notch widths over 100 μm to an average of 5.64 MPa√m for FIB‐notched specimens.

In round robin testing on 3Y‐TZP specimens (sourced from Tosoh powder and sintered at 1,510°C for 2 h with an average grain size of 0.45 μm), the results of 33 tests from multiple laboratories yielded a fracture toughness of 4.36 ± 0.44 MPa√m using the SCF method (Quinn et al., 1994). Another round robin study was performed on the same lot of 3Y‐TZP bars using the SEVNB method, which yielded a fracture toughness of 5.34 ± 0.65 MPa√m from 35 tests performed at seven laboratories (Kübler, 2000). This later round robin study, referenced in ISO 23146 (2008), reported notch widths ranging between 2 and 18 μm, and that none of the round robin participants were able to achieve a notch width less than about twice the size of the larger diameter grains of the lot of 3Y‐TZP, or about 1.6 μm (Kübler,1999, 2000). This observation combined with the lower average SCF fracture toughness results led to the conclusion that the SEVNB method should not be used for 3Y‐TZP, as it delivers overestimated fracture toughness values for this fine‐grained material. In the current study, we achieved the goal of creating notch widths <100 nm in 3Y‐TZP, which is less than the average grain size, resulting in fracture toughness values of 5.64 ± 1.14 MPa√m. However, it is not possible to make conclusive statements about the comparability of this method with other standard methods, such as the SCF and SEPB methods, without further testing.

Recent work (Turon‐Vinas & Anglada, 2014, 2018) noted the difficulty in producing notch tip radii of <1 μm in 3Y‐TZP, and reported on addressing this problem with the use of ultra‐short pulsed laser ablation (UPLA) to introduce a sharp crack in the material. Beam specimens of 3 mm × 4 mm × 45 mm were produced from 3Y‐TZP powder with a 3 mol% yttria (Tosoh, grade TZ‐3YSB‐E) that was cold uniaxially pressed and then sintered at 1,450°C for 1 h. It was shown that the shallow notch produced by UPLA achieved a notch tip radius in the submicron range with a depth of about 30 μm (Turon‐Vinas & Anglada, 2014, 2018). A region of microcracks in front of the notch, which extended an additional ~10–20 μm in length by ~4 μm in width, were included in the total crack length, resulting in a fracture toughness of 4.1 ± 0.4 MPa√m (Turon‐Vinas & Anglada, 2014, 2018). Similar to the FIB‐notched and saw blade surfaces in our study, the UPLA notch faces and fracture surfaces of the microcracked zone exhibited no monoclinic phase, while a small amount of monoclinic phase was discerned on the fracture surfaces of the 3Y‐TZP specimens, and about 68% monoclinic phase was measured on the fracture surfaces of similarly prepared 12Ce‐TZP specimens (Turon‐Vinas & Anglada, 2014, 2018).

In the FIB‐notching study by Fett et al. (2008), the authors developed a bending bar test specimen that allowed fracture toughness measurements to be made on very sharp and short notches to obtain a fracture toughness value for a Ce‐ZrO_2_ ceramic of 5.9 MPa√m. To make the appropriate stress intensity factor calculations, finite element computations were performed to determine the geometric function. In the present study, sharp and short FIB‐notches were placed at the bottom of starter notches in standard SEVNB specimens, and an assumption is made that the effective crack length equals the notch depth for the equation used to calculate fracture toughness. For the starter notch plus the FIB‐notch to behave as a straight long crack, the length of the FIB‐notch needs to be long enough so that the root radius of the sawblade‐notch does not strongly influence the crack tip stress. Annex C of ISO 23146 (2008) and Fett (1995, 1999) described the true fracture toughness for an edge crack in front of a finite notch as follows:

$$K_{\mathrm{Ic}}^{*}=K_{\mathrm{Ic}}∙\tan h\left(2.243\sqrt{\frac{\mathrm{\delta a}}{R}}\right)$$

where *K*_Ic_ is the experimental fracture toughness, *δa* the depth of the crack in front of the notch, and *R* the notch root radius. If the ratio of $K_{\mathrm{Ic}}^{*}$/*K*_IC_ is given an acceptable value of 0.95 (Fischer et al., 2008) and the sawblade‐notch root radius of ~75 μm was used, then a “critical crack length” can be calculated for *δa*, showing that the FIB‐notches of ~5 μm were not deep enough to overcome the crack tip stress of the blunt starter notch, and presumably resulted in an overestimated experimental fracture toughness. A longer FIB‐notch and/or sharper starter notch will minimize the influence of the crack tip stress of the notch root radius. For instance, it can be calculated that for a 5 μm FIB‐notch, the starter root notch radius needs to be reduced to below 10 μm, which is achievable, in order to treat the starter notch and FIB‐notch as a straight long crack.

The fracture toughness values for 3Y‐TZP vary by manufacturer, milling, and sintering process, among other factors, making it complicated to directly compare the fracture toughness values in this study to those reported in the literature. It is, however, evident that FIB‐milling reduced fracture toughness values by more than 3 MPa√m compared to the saw blade‐notched specimens without FIB‐notches. The post‐mortem examination readily identified that fractures started precisely from the FIB‐notches. The FIB‐notches are uniform throughout the thickness with consistent depth, making them a model system for fracture analysis.

The tetragonal‐to‐monoclinic phase transformation that occurs in the fracture process, forms a thin layer of monoclinic phase on the surface. We hypothesize that the transformation toughening, rather than being homogenous across the fracture path, is inhomogeneous and localized. The bright spots consisting of a large amount of monoclinic phase may serve as pinpoints retarding fracture propagation, thus enhancing the resistance to fracture. Gogotsi (2003) observed the slight presence of monoclinic phase in the fracture surface. However, it is unclear whether or not the monoclinic phase is fracture induced, as the non‐fractured surface also showed some monoclinic phase (Gogotsi, 2003) . In our report, the monoclinic phase was created by the fracture, while the un‐fractured surface had no detectable monoclinic phase. The exact thickness of monoclinic phase is unknown due to the limitations of Raman microscopy.

A drawback of FIB‐milling is the irradiation damage induced by energetic gallium ion bombardment (Mayer, Giannuzzi, Kamino, & Michael, 2007). It has been reported that a 20–30 nm thick amorphous layer can be formed on the surface of samples as a result of this ion bombardment (Kato, 2004). However, it is unclear how this nano‐amorphous layer influenced the fracture toughness values of the bulk material. Another major concern was whether FIB‐milling created any tetragonal‐to‐monoclinic phase transformation. In fact, the Raman analysis of the FIB‐notched surfaces from the fractured specimens showed that the ion beam irradiation did not introduce detectable levels of monoclinic phase.

## 5 CONCLUSION

We developed a highly repeatable method to fabricate nanometer‐sized notches in 3Y‐TZP that are consistent through the width of a standard specimen using focused ion beam milling. The FIB‐notches demonstrated consistent height across the entire width of the specimens, and the openings at the notch tips were consistently <100 nm, which is smaller than the grain size of the 3Y‐TZP material. Fracture analysis demonstrated that the fractures started exactly from the FIB‐notches, and the fracture toughness was measured to be 5.64 ± 1.14 MPa√m, which is considerably lower than the fracture toughness value generated from the saw blade‐notched specimens without the FIB‐notches. For the milled specimens, the fracture origin can be readily measured post‐mortem below the FIB‐notches. While the FIB‐milling process inevitably creates an amorphous nanolayer, the process does not generate measureable levels of monoclinic phase, as determined by Raman analysis. FIB has become relatively accessible in both industry and academics and, thus, FIB‐milling may be promising as a technique to generate nanometer‐sized notches in ceramics for fracture toughness measurements. Further testing is needed to fully understand the significance of the geometry of the FIB‐notch on results, and the comparability of this method with other standard methods, such as the SCF and SEPB methods.
